# A new ensemble-based targeted observational method and its application in the TPOS 2020

**DOI:** 10.1093/nsr/nwad231

**Published:** 2023-09-02

**Authors:** Weixun Rao, Youmin Tang, Yanling Wu, Zheqi Shen, Xiangzhou Song, Xiaojing Li, Tao Lian, Dake Chen, Feng Zhou

**Affiliations:** College of Oceanography, Hohai University, Nanjing210024, China; College of Oceanography, Hohai University, Nanjing210024, China; Faculty of Environment, University of Northern British Columbia, Prince George, British ColumbiaV2N 4Z9, Canada; College of Oceanography, Hohai University, Nanjing210024, China; Key Laboratory of Marine Hazards Forecasting, Ministry of Natural Resources, Hohai University, Nanjing210024, China; Innovation Group of Earth System Model, Southern Marine Science and Engineering Guangdong Laboratory (Zhuhai), Zhuhai519015, China; College of Oceanography, Hohai University, Nanjing210024, China; Key Laboratory of Marine Hazards Forecasting, Ministry of Natural Resources, Hohai University, Nanjing210024, China; Innovation Group of Earth System Model, Southern Marine Science and Engineering Guangdong Laboratory (Zhuhai), Zhuhai519015, China; College of Oceanography, Hohai University, Nanjing210024, China; Key Laboratory of Marine Hazards Forecasting, Ministry of Natural Resources, Hohai University, Nanjing210024, China; Innovation Group of Earth System Model, Southern Marine Science and Engineering Guangdong Laboratory (Zhuhai), Zhuhai519015, China; State Key Laboratory of Satellite Ocean Environment Dynamics, Second Institute of Oceanography, Hangzhou310012, China; Innovation Group of Earth System Model, Southern Marine Science and Engineering Guangdong Laboratory (Zhuhai), Zhuhai519015, China; State Key Laboratory of Satellite Ocean Environment Dynamics, Second Institute of Oceanography, Hangzhou310012, China; Innovation Group of Earth System Model, Southern Marine Science and Engineering Guangdong Laboratory (Zhuhai), Zhuhai519015, China; State Key Laboratory of Satellite Ocean Environment Dynamics, Second Institute of Oceanography, Hangzhou310012, China; State Key Laboratory of Satellite Ocean Environment Dynamics, Second Institute of Oceanography, Hangzhou310012, China

**Keywords:** targeted observation, ENSO, TPOS, data assimilation

## Abstract

Ensemble Kalman filter-based targeted observation is one of the best methods for determining the optimal observational array for oceanic buoy deployment. This study proposes a new algorithm suitable for a ‘cross-region and cross-variable’ approach by introducing a projection operator into the optimization process. A targeted observational analysis was conducted for El Niño–Southern Oscillation (ENSO) events in the tropical western Pacific for the Tropical Pacific Observation System (TPOS) 2020. The prediction target was at the Niño 3.4 region and the first 10 optimal observational sites detected reduced initial uncertainties by 70%, with the best observational array located where the Rossby wave signal dominates. At the vertical level, the most significant contribution was derived from observations near the thermocline. This study provides insights into understanding ENSO-related variability and offers a practical approach to designing an optimal mooring array. It serves as a scientific guidance for designing a TPOS observation network.

## Teaser

A new targeted observational algorithm was developed to optimize prediction targets across various regions and variables. This approach was utilized to design an optimal ENSO monitoring array in the TPOS 2020 project.

## INTRODUCTION

Target observation, also known as adaptive observation, is an observation strategy that was developed in the late 1990s [1]. The main concept is to identify an optimal observational location or region and increase observations to achieve maximum forecast improvement for the forecast object by reducing uncertainty in the initial conditions [1]. Initial uncertainty is usually derived from coarse observations assimilated into numerical models. However, a large-scale increase in observations is costly and challenging to implement. The key to overcoming this problem is increasing the number of adequately placed observations. After observations in some essential areas are encrypted and assimilated into the model, the prediction skill can be significantly improved [1,2]. Therefore, it is important to develop an effective and efficient target observation strategy for optimal observational arrays.

There are currently two methods for determining optimal observational locations [3,4]. One is to seek the most significant growth in initial errors, which are assumed to affect the prediction. This method operates under the control theory framework by optimizing a cost function with the constraint of the original dynamic systems. This poses the hypothesis of the perfect model—that is, the most significant growth of prediction errors is completely dominated by the initial uncertainty, which can be eliminated by assimilation [2]. Such methods include breeding vectors [5], singular vectors [6] and conditional non-linear optimal perturbations [7].

The second method was developed based on ensemble assimilation technology. This method attempts to find the optimal observational locations in conjunction with a sequential assimilation method, including the ensemble transform Kalman filter [8], ensemble sensitivity analysis [3,9] and particle filter [10], so that assimilating the optimal observations can result in a minimum subsequent prediction error variance. This is implemented by imposing to select the optimal observations one by one gradually, since it has been theoretically proven that serial assimilation is equivalent to parallel assimilation of all observations at one time, given that the observations are non-covariant [11]. This ensures computational efficiency and economical computing resources.

Typically, the second method determines the optimal location in a grid-by-grid format—a feature that is well suited for the actual array implementation. However, the sequential assimilation method updates the background error covariance at each assimilation step, meaning that the optimal observational location determined is flow-dependent [4]. This poses challenges in the design of long-term observational networks. To address this, Sakov and Oke in 2008 (SO08 hereafter) [12] developed a method based on Ensemble Optimal Interpolation to design an optimal array for the seasonal prediction of sea-level height over the tropical Indian Ocean, in which the background error covariance was flow-independently estimated by using long-term integration. Moreover, the uncertainty in the initial field is quantized by the trace of the background error covariance matrix and targeted observations can be identified by minimizing the trace of the background error covariance matrix [11]. Notably, SO08 identifies the n-*th* optimal observation based on the assimilation of the first (*n* – 1) optimal sites, allowing the quantification of the relative and cumulative contribution of the optimal observations. Liu *et al.* [13] used this method to perform an array design for the northwestern tropical Pacific Ocean circulation prediction of the timescale at intra-seasonal and interannual variation in the northwest tropical Pacific.

SO08 has advantages of simplicity and high computational efficiency; however, it searches only for optimal sites in the same area as the prediction domain. It also confines the observational variables to the same prediction variables. These shortcomings limit the further application of SO08 in more general situations. For example, the Tropical Pacific Observation System (TPOS) 2020 project mainly aims to understand and predict the El Niño–Southern Oscillation (ENSO) but its focus is on repairing the observational array with multi-layer observations in the western Pacific (WP) region. Thus, the first objective of the current study is to extend the SO08 algorithm and develop a new approach allowing the observational region/variables to differ from the prediction target—that is, a new algorithm suitable for the ‘cross-region and cross-variable’ approach. In particular, the new algorithm can determine the optimal mooring location in the framework of a 3D observational network. Hou *et al.* [14] developed the targeted observation method based on the particle filter [10], making it suitable for ‘cross-region and cross-variable’ scenarios. However, the current algorithm of particle filter identifies all the optimal observational sites independently using the original prediction ensemble, without considering any assimilation of previously identified optimal observations. Consequently, it does not provide a quantification of the individual and cumulative improvement of the optimal observations.

The ENSO is the most influential interannual oscillation involving coupled atmosphere–ocean processes [15]. The TPOS has played an important role in understanding, monitoring and forecasting the ENSO [16]. However, owing to logistical reasons, the mooring array in the WP region has significantly deteriorated [17]. Relevant research has shown that damage to the observational array affects our ability to model and predict the ENSO [18,19]. Further, the diversities and complexities of the ENSO have long been recognized as a significant obstacle to ENSO predictions [20,21]. The occurrence of multiyear La Niña events in recent years has also posed a considerable challenge for simulating and predicting the ENSO [22,23]. To address these challenges, the new international project, the TPOS 2020, aims to redesign and optimize the tropical Pacific observational network. One of the key points of the TPOS 2020 is to restore and improve the WP array to enable more comprehensive description, understanding and predictions of the ENSO [17]. Moreover, China has recently proposed a regional observation program to contribute to the TPOS. The task of this program is to deploy mooring buoys in the WP region, although the deployment locations still require further scientific demonstration [24]. Thus, the second objective of our current study was to apply the developed algorithm to design an optimal mooring array in the WP region for ENSO predictions.

## RESULTS

In the tropical Pacific, the westward equatorial current forced by easterly winds produces higher sea-surface temperatures (SST) in the WP region, resulting in a deeper thermocline in the west than in the east [25]. The thermocline depth in the tropical WP is mainly within the range of 100–150 m, while it is only ∼50 m in the eastern Pacific (Supplementary Fig. 1a). According to the standard deviation (STD) of the temperature (Supplementary Fig. 1b), we selected five layers with significant temperature variations for the chosen depths in a mooring array, which were 5, 100, 120, 150 and 200 m. Moreover, in the vertical direction, there were notable ocean temperature variations near the thermocline and these areas were significantly affected by ocean dynamics [26].

We conducted a sensitivity experiment by selecting five layers of the upper ocean (i.e. 5, 50, 100, 150 and 200 m) for the same targeted observational analysis. The results showed that the most important optimal locations in the horizontal direction remained the same. However, they resulted in a smaller decrease in the analysis error variance when assimilated compared with that of the aforementioned optimal depths. Significant ocean temperature variability mainly occurred near the thermocline, which significantly affected ENSO variability. Thus, the ENSO is more sensitive to observations at depths with greater temperature gradients.

### Observational array designed by surface data

**Figure 1.**
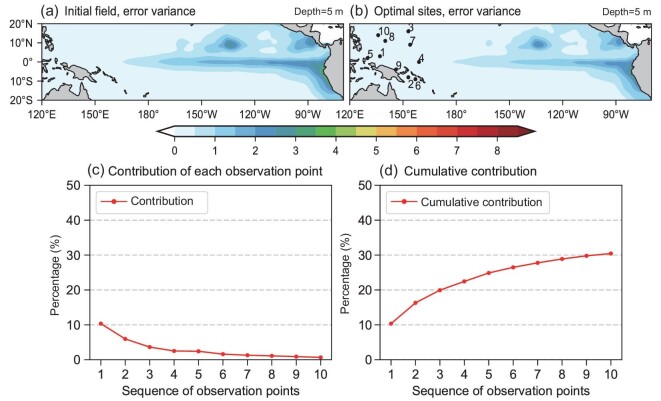
(a) The initial error variance $( {^ \circ {{\mathrm{C}}}^2} )$ of SST and (b) the theoretical analysis error variance $( {^ \circ {{\mathrm{C}}}^2} )$ of SST using the optimal array design based on sea-surface data. The marked points and numbers in (b) denote the site locations and ranking of each optimal observation (i.e. the location marked ‘1’ is the best location). (c) The percentage reduction of the initial error variance of the Niño3.4 SSTA using the *n*-th optimal observational location from the observational array design based on sea-surface data and (d) the cumulative reduction of the first *n* observations.

We sought the optimal observational locations for SST to optimize the Niño 3.4 SST anomalies (SSTA). The optimal observations were restricted to the WP region (i.e. 120°E–160°E and 20°S–20°N) and the ensemble members were generated by the monthly SSTA from 1980 to 2020. The results are shown in Fig. 1, which presents the initial and theoretical analysis error variance of SST. The locations of the 10 identified optimal observational sites are marked by dots in Fig. 1b, accompanied by ranked numbers from 1 to 10. In Fig. 1b, the number 1 indicates the optimal site that can result in the largest reduction in analysis error variance of the Niño3.4 region during assimilation, followed by numbers 2, 3 and so on.

A comparison between Fig. 1a and b revealed that the 10 optimal observations could not result in a significant reduction in the initial error variance. There were still great uncertainties in the tropical central and eastern Pacific after assimilation (Fig. 1b), suggesting that assimilation of SST in the WP region may not significantly improve ENSO prediction. This is consistent with previous studies that argued that subsurface temperature, rather than SST, in the WP region plays a major role in ENSO variability [26–31]. Figure 1c shows the percentage reduction in the background error variance of the Niño3.4 SSTA for each assimilated optimal site. Figure 1d is similar to Fig. 1c but indicates the cumulative percentage reduction in the first *n* sites. As shown in Fig. 1d, assimilation of the optimal SST observations in the WP region can reduce the initial error variance of the Niño3.4 SST by nearly 30%. Among all 10 optimal sites, the first observation provided a 10% reduction, followed by a 6% reduction. The remaining observations individually resulted in a reduction of <5%.

### Optimal observational array designed by multi-layer data

We also carried out a targeted observational analysis for ENSO predictions using the cross-region and cross-variable method to determine the optimal mooring array of ocean temperatures. For sensitive analysis, we also used the SODA data to repeat the analysis [32] and obtained similar results (not shown), indicating that the results reported here are robust and little data-dependent.

**Figure 2.**
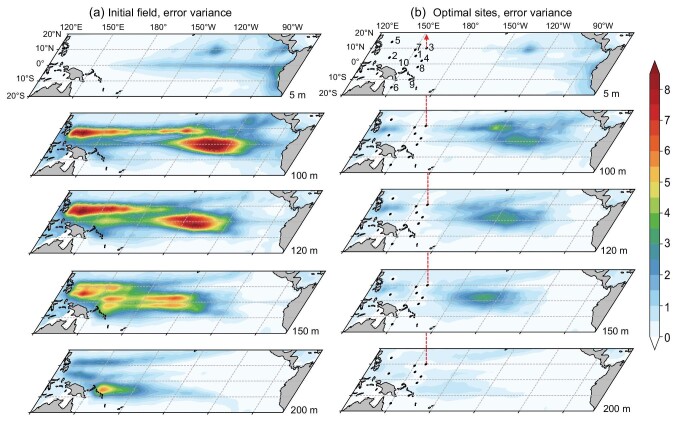
(a) The initial error variance $( {^ \circ {{\mathrm{C}}}^2} )$ of ocean temperature and (b) the theoretical analysis error variance $( {^ \circ {{\mathrm{C}}}^2} )$ at the depths of 5, 100, 120, 150 and 200 m. The marked points and numbers in (b) denote the mooring locations and ranking of each optimal site.

We first evaluated the initial uncertainty of the ocean temperature at the selected five layers and the initial error variance of the ocean temperature in each layer is shown in Fig. 2a. For the sea surface, the most significant error variance occurred in the cold tongue, extending westward from the coast of Peru with a sharp front in the central Pacific. The maximum SSTA variation in ENSO events usually occurs in these regions [33]. For the subsurface, there were two areas with the maximum temperature error variance, which were located near 7°N of the WP region and the equatorial central Pacific at a depth of ∼100–150 m. These are the regions where the main thermocline was located. Wyrtki [27] found that the volume of warm water (WWV) (equivalent heat content) in the tropical Pacific increased gradually prior to an El Niño event and only fell precipitously during the event. The main thermocline controls the temperature of the upwelling water in the eastern equatorial Pacific by changing the WWV—that is, the deeper main thermocline causes the upwelling of the warmer water and vice versa, which plays an important dynamic role in the oscillation of the ENSO cycle as argued by a classic mechanism of the ENSO called the ‘recharge oscillator’ [29,30]. Therefore, there is pronounced interannual variability associated with the ENSO near the main thermocline in the subsurface ocean, which results in significant sensitivity to initial uncertainties.

We obtained an optimal observational array with 10 observational sites in the WP using the proposed algorithm (Fig. 2b). The marked points show the locations of the array sites, indicated by numbers 1 to 10. The analysis error variance of the temperature for each layer was theoretically estimated after assimilating these optimal observations, indicating a reduction in analysis error variance for each layer. Compared with Fig. 2a, the large initial error variance in the eastern Pacific is also effectively reduced in Fig 2b. This indicates that 3D optimal observations of the WP can effectively reduce the initial errors of thermal fields in the entire tropical Pacific.

The highest three optimal sites are on both sides near 7°N, where the subsurface ocean temperature had the most significant initial error variance (Fig. 2a). According to the distribution of ocean temperature STD, the most significant initial errors were observed in the subsurface of the WP region (Supplementary Fig. 1). This suggests that this area is sensitive to monitoring ENSO events and was interpreted well by the importance of off-equator physical processes in the WP [31,34–36]. From the perspective of ocean-wave dynamics, the key dynamical process during the development and formation of ENSO events was confined to within 8° of the equator in the form of the symmetric Rossby modes and the equatorial Kelvin signals, as described by the delayed-oscillator mechanism [28].

**Figure 3.**
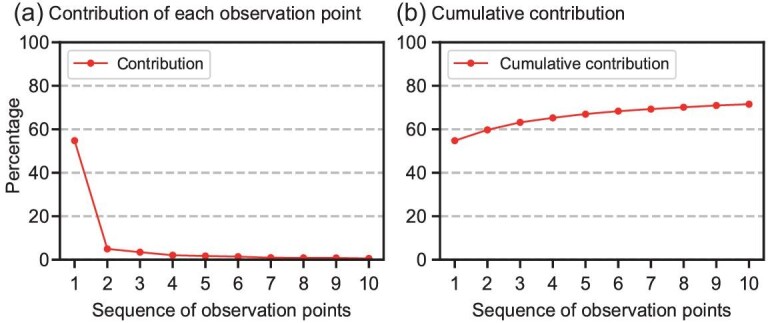
(a) The percentage reduction of the initial error variance of the Niño3.4 SSTA using the *n*-th optimal observational location and (b) the cumulative reduction of the first *n* observations.

The theoretical reductions in the background error variance of the Niño3.4 SSTA obtained by assimilating the optimal observations are shown in Fig. 3. After assimilating the five-layer profile of the temperature observation of the first site, the initial uncertainty was dramatically reduced by 54% (Fig. 3a). The 10 optimal profiles can theoretically reduce the initial error variance by >70% (Fig. 3b), which is much better than the observational array based on the surface (Fig. 1d), indicating that multi-layer observations are important for ENSO prediction. The initial error variance reduced by the observations was relatively limited after the second site. This may be because all the locations of the observational array are confined in the WP and the observational information of subsequent locations is highly related to that of the first location.

**Figure 4.**
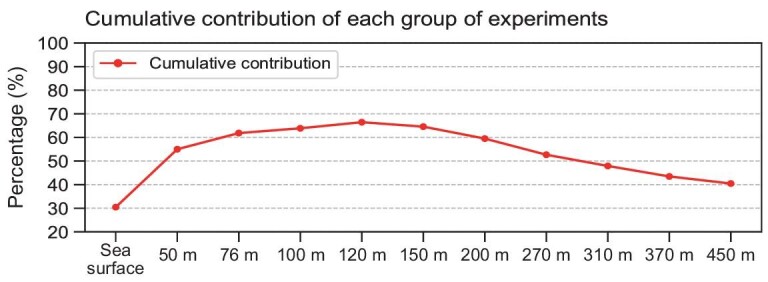
The percentage reduction in the initial error variance of the Niño3.4 SSTA using two observations, which were from the surface and a certain depth in the subsurface at the first mooring location shown in Fig. 2b. The first value denotes the percentage reduction of the initial error variance of the Niño3.4 SSTA by only using the surface observation.

We also examined which layer of observation made the most significant contribution in terms of reduction in the initial background error variance. The first optimal observational site in Fig. 2b was selected for this analysis. Only two observations were assimilated into the initial field—one from the surface and the other from the subsurface layer. In other words, the value corresponding to 50 m is the percentage reduction in the initial errors after assimilating the temperature anomalies from the sea surface and a depth of 50 m. The reduction in the initial error variance is presented in Fig. 4. It is indicated that the error reduction was the largest when the observation at a depth of 120 m was assimilated, which is just around the position of the mean thermocline. This confirms the importance of observations near the thermocline and the necessity for observational deployment at this layer.

The highest three influential levels shown in Fig. 4 are 100, 120 and 150 m, respectively, which are around the depth of the thermocline with significant ocean temperature variability in the WP. The thermocline of the WP is important for ENSO progression. During ENSO events, there is an active Rossby wave propagation off-equator [37]. Previous studies have indicated that Rossby waves modulate the depth of the thermocline along the propagation path eastward from the WP region [38]. Meinen and McPhaden [39] also confirmed that the WWV [27] associated with thermocline depth is a useful predictor of ENSO events. We found that the first three sites of the optimal array coincided with the propagation path of the Rossby waves. Therefore, observations at the thermocline near 7°N are critical for ENSO predictions.

**Figure 5.**
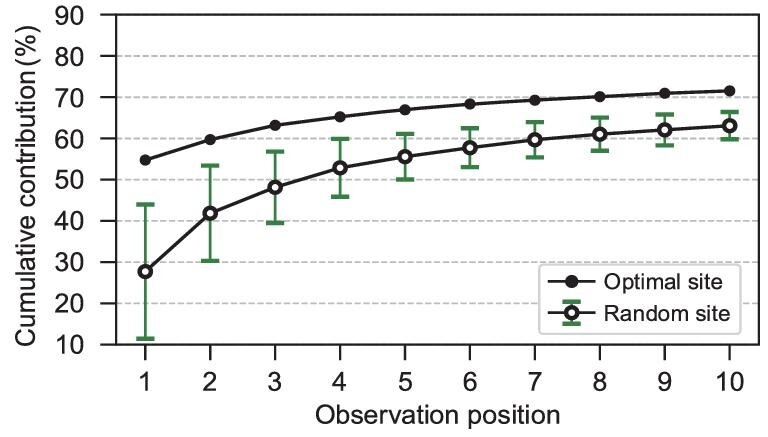
The cumulative percentage reduction of the initial error variance of the Niño3.4 SSTA contributed by the first *n*-th observational site using the optimal array (10 sites, black dot) and 100 random arrays (hollow dot). The black dot plot is the same as in Fig. 3b. The hatched bars indicate the corresponding STD of the cumulative percentage reduction contributed by the first *n*-th observational location in 100 random arrays.

A statistical significance test of the optimal observational array was conducted using the Monte Carlo method. We randomly chose 10 locations of five-layer temperatures in the WP to form an observational array and calculated the cumulative percentage reduction of the error variance of the Niño 3.4 SSTA while they were assimilated. This procedure was repeated 100 times. The mean and STD of the cumulative reduction are shown as hollow dots and hatched bars in Fig. 5, respectively. The effect of these random arrays had great uncertainty, particularly when the number of observation locations is limited. Moreover, the performance of the optimal observations was significantly better than that of random observations. This result indicates effective and significant improvement by the optimal observational array for its ENSO monitoring ability.

In the above analysis, we used monthly mean data to seek the optimal observational sites. This is because we aim at detecting steady and long-term observing sites for ENSO prediction, which is expected to filter out noise contamination in the analysis. It has been argued that the low-frequency signal is the key to ENSO prediction. In addition, the noise is often treated as white noise, having a statistical covariance value of zero with the low-frequency signal.

However, it is interesting to explore how the above results are sensitive to the noise level. To investigate this issue, we repeated all analyses of targeted observation but used noisy data, which are obtained by perturbing noise to the raw ensemble (i.e. the matrix ${{{\bf A}}}_{{\bf b}}$ in Equation (5)). We carried out three experiments, adding white noise with a variance of 10%, 20% and 30% of the monthly ocean temperature, respectively.

**Figure 6.**
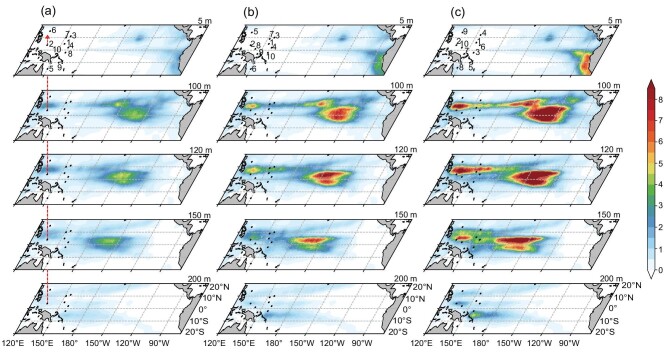
(a–c) The theoretical analysis error variance $( {^ \circ {{\mathrm{C}}}^2} )$ at the depths of 5, 100, 120, 150 and 200 m. The marked points and numbers denote the mooring locations and ranking of each optimal site. This is the same as in Fig. 2b but with white noise added to the initial prediction ensemble. The variance of the white noise is 10%, 20% and 30% of the monthly ocean temperature variance in (a), (b) and (c), respectively.

The results show that when the noise is added to the raw data, the initial error variance increases compared with the raw data shown in Fig. 2. The amplitude of the initial error variance depends on the noise level (not shown). Correspondingly, the analysis error variance also increases, as shown in Fig. 6. However, the spatial patterns of error variance are very similar to those from the raw data, as in Fig. 2b.

The optimal observational sites detected from the noisy data are also shown in Fig. 6. A comparison with the raw data reveals that the first four optimal observation sites, accounting for >60% reduction of error variance, remain unchanged for the noisy data of 10% and 20% variance (Fig. 6a and b). When the noise level increases to 30%, the first two optimal sites, explaining a 50% reduction of error variance, remain unchanged and most of the remaining sites just exchange positions (Fig. 6c). Thus, compared with the raw data, the optimal sites of noisy data that make the main contributions to the reduction in error variance remain largely the same. Most optimal sites are located at the off-equatorial region within 7°N latitude, where the Rossby wave influences are prevalent.

As the noise level increases, the total improvements in the 10 optimal profiles decrease slightly. For the raw data, 10 optimal sites could theoretically reduce the initial error variance by 72% (Fig. 3b), in contrast to 70%, 66% and 61% for the three experiments (Supplementary Fig. 2), respectively.

Overall, the above sensitivity experiments indicate that the main results presented here are considerably robust, even though the noisy level is increased to 30% of the raw data variance.

## SUMMARY AND DISCUSSION

In this study, we developed a new targeted observational algorithm suitable for a ‘cross-region and cross-variable’ approach, based on the ensemble data assimilation theory. This method determines optimal observations by minimizing the analysis error variance. Compared with the commonly used approach proposed by SO08, which assumes that the observation and the prediction area are the same, our new algorithm could optimize the prediction target in a specific region that is different from the observational domain, referred to as the ‘cross-region’. By introducing a projection operator, this method could also optimize a prediction variable different from the observed ones, indicated as a ‘cross-variable’. This method can determine the observations that bring the most significant reduction in analysis error variance and quantify the benefits resulting from the assimilation of each optimal observation.

Based on the requirements of the TPOS 2020 project, we used this method to design an optimal observational array in the WP region for long-term monitoring of ENSO events. Multi-layer ocean temperatures in the upper ocean were used as observational variables and the SSTA in the Niño 3.4 region was used as the prediction target. The results showed that the first 10 multi-layer optimal observational arrays could theoretically reduce the initial error variance by nearly 70%, which is much better than that obtained using only the surface observation. The most effective optimal observational sites were located near 7°N, where near equatorial Rossby wave influences are prevalent [28]. Through sensitivity experiments, we also determined that, in the vertical direction, observations near the thermocline are the most important in reducing the background error variance. Thermocline feedback can cause variability in the WWV [29,30], which is usually considered a precursor of ENSO events [39]. In addition, the atmospheric response caused by thermocline adjustment is an important part of the negative feedback of the ENSO [31,40]. Therefore, observations near the thermocline are beneficial for monitoring and predicting ENSO events. This targeted observational analysis emphasizes the importance of temperature variability in the WP region with active Rossby waves and the thermocline region, which is also consistent with the ENSO dynamic framework, such as the delayed oscillator and recharge oscillator [26,28–30]. The results are also consistent with other target observation work, highlighting the importance of the WP region during the formation and development of ENSO events [41]. The eastern equatorial Pacific is also emphasized when designing optimal observation sites across the entire tropical Pacific [14,41].

The mooring array can provide steady observations with high temporal resolution, ranging from hourly to daily measurements. For this study, monthly mean data were conventionally used to filter out high-frequency noise [42]. To examine the potential influences of noise, we conducted sensitive experiments by incorporating white noise into the initial field. The results suggested that the primary characteristics of the targeted observation remained largely unaffected. Some optimal sites located in offshore regions may look odd, but they could be reminiscent of ENSO dynamics and physical processes, given that the reflection of Rossby waves at the western boundary plays an important role in the ENSO dynamical mechanism. In addition, one should take caution in interpreting some sites with small contributions to the error variance reduction, which may be a product of mathematical optimization rather than a physical solution. When deploying the array at sites with a limited contribution, thoughtful planning may be required in real-world applications.

In future work, an observing system simulation experiment will be conducted to verify the effectiveness and optimality of the ‘cross-region and cross-variable’ method. It is worth noting that this method uses long-term integration of climate models or reanalysis data to estimate the background error covariance, ignoring the flow-dependent nature of system states. Therefore, it may be more realistic to construct a flow-dependent ensemble to estimate the uncertainty in the initial field, although this will bring challenges to designing a long-term stable oceanic mooring array, which is underway.

In the TPOS 2020 project, the regional observation program undertaken by China urgently needs to design a buoy layout scheme for the WP region. Based on solving scientific problems, this study proposes alternatives to improve the TPOS, which is expected to provide technical support for China's observing system design. In addition, the ensemble Kalman filter (EnKF) theory assumes that both model and observation errors follow a Gaussian distribution. Therefore, the proposed method based on the EnKF theory is more suitable for dynamic systems in which non-linear and non-Gaussian processes are not dominant. With the widespread application of ensemble data assimilation technology, the proposed targeted observational method is expected to be widely used in establishing real-time observational networks.

## METHODS

We assumed a state vector ${{\boldsymbol x}} \in {\mathbb{R}}^n$ to exist in the discrete system, where *n* represents the size of the variables in three dimensions. The background error covariance matrix representing the uncertainty of the model state is ${{{\bf P}}}^{{\bf b}} \in {\mathbb{R}}^{n \times n}$. The analysis error covariance matrix ${{{\bf P}}}^{{\bf a}}$ can be derived by assimilating observations with error covariance ${{\bf R}}$. According to the Kalman filter theory,


(1) \begin{eqnarray*}{{{\bf P}}}^{{\bf a}} = [ {{{\bf I}} - {{{\bf P}}}^{{\bf b}}{{{\bf H}}}^{\mathrm{T}}{{\left( {{{\bf H}}{{{\bf P}}}^{{\bf b}}{{{\bf H}}}^{\mathrm{T}} + {{\bf R}}} \right)}}^{ - 1}{{\bf H}}} ]{{{\bf P}}}^{{\bf b}},\end{eqnarray*}

where ${{\bf I}}$ denotes the identity matrix and superscript ${\mathrm{T}}$ denotes a matrix transpose. ${{\bf H}}$ denotes the observation matrix, including information on the observational sites.

As SO08 proposed, the optimal observations are indicated by an observational matrix ${{{\bf H}}}^{\rm opt}$, which minimizes the trace of ${{{\bf P}}}^{{\bf a}}$ among all observations, as follows:


(2) \begin{eqnarray*}{{{\bf H}}}^{\rm opt} = \arg \mathop {{\mathrm{min}}}\limits_{\left\{ {{\bf H}} \right\}} {\mathrm{trace}}\left( {{{{\bf P}}}^{{\bf a}}} \right).\end{eqnarray*}

However, Equation (2) implies that the SO08 method uses all variables over the entire region as the prediction target, with which the TPOS 2020 can hardly meet the demand for ENSO target observations. Specifically, the observed variable in this study is the ocean temperature at *k* representative depths and the prediction targets are the Niño 3.4 SSTA. Hence, we attempted to improve the methodology and develop a ‘cross-region’ and ‘cross-variable’ algorithm by introducing a projection operator. If ${\boldsymbol{u}} \in {\mathbb{R}}^s$ is a vector of the SSTA at grids spanning the Niño3.4 region (assuming its size is *s*), we define a matrix ${{\bf Lr}} \in {\mathbb{R}}^{s \times n}$ that could project the full state vector ${\boldsymbol{x}}$ to its subvector ${\boldsymbol{u}}$. ${{\bf Lr}}$ can be easily generated as follows. Assuming ${i}_1,{i}_2, \ldots ,{i}_s$ are indices of the elements of ${\boldsymbol{u}}$ in ${\boldsymbol{x}}$, ${{\bf Lr}}$ can be derived by selecting the rows with indices ${i}_1,{i}_2, \ldots ,{i}_s$ from the identity matrix. In other words, ${{\bf Lr}}$ can be expressed by ${[ {{{\boldsymbol{e}}}_{{{\boldsymbol{i}}}_1},{{\boldsymbol{e}}}_{{{\boldsymbol{i}}}_2}, \cdots ,{{\boldsymbol{e}}}_{{{\boldsymbol{i}}}_{\boldsymbol{k}}}, \cdots ,{{\boldsymbol{e}}}_{{{\boldsymbol{i}}}_{\boldsymbol{s}}}} ]}^{{\bf T}}$, where ${{\boldsymbol{e}}}_{{{\boldsymbol{i}}}_{\boldsymbol{k}}}$ represents a canonical basis column vector with a value of 1 at the ${i}_k$-th position and 0 for all other elements.

In this case, one optimal location of the proposed method could be determined as:


(3) \begin{eqnarray*}{{{\bf H}}}^{{\mathrm{opt}}} = \arg \mathop {\min }\limits_{\left\{ {{\bf H}} \right\}} {\mathrm{trace}}\left\{ {{{\bf Lr}}{{{\bf P}}}^{{\bf a}}{{\bf L}}{{{\bf r}}}^{\mathrm{T}}} \right\}.\end{eqnarray*}

The solution of Equations (1) and (3) could be written as:


(4) \begin{eqnarray*}
{{{\bf H}}}^{{\mathrm{opt}}} &=& \arg \mathop {\max }\limits_{\left\{ {{\bf H}} \right\}} {\mathrm{trace}}\big\{ {{\bf Lr}}\big[ {{{\bf P}}}^{{\bf b}}{{{\bf H}}}^{\mathrm{T}} ( {{{\bf H}}{{{\bf P}}}^{{\bf b}}{{{\bf H}}}^{\mathrm{T}} }\\
&& +\, {{{\bf R}}}_{{\bf H}} )^{ - 1} {{\bf H}} {{{\bf P}}}^{{\bf b}} \big] {{\bf L}} {{{\bf r}}}^{\mathrm{T}} \big\},
\end{eqnarray*}

where ${{{\bf R}}}_{{\bf H}} \in {\mathbb{R}}^{s \times s}$ is the observation error covariance related to the observation matrix ${{\bf H}}$. It is assumed that the observation error variance is not related in space, hence the serial assimilation is equivalent to simultaneously assimilating all observations at once [11].

In practice, we construct ${{{\bf P}}}^{{\bf b}}$ using a representative ensemble of system state anomalies ${{{\bf A}}}_{{\bf b}}$:


(5) \begin{eqnarray*}{{{\bf P}}}^{\bf{b}} = {{{\bf A}}}_{{\bf b}} \times {{{\bf A}}}_{{\bf b}}^{\mathrm{T}}/m,\end{eqnarray*}

where ${{\bf A}}_{\bf b}$ = [${\boldsymbol{x}}_1^{\prime}$,$\ {\boldsymbol{x}}_2^{\prime}$, …, ${\boldsymbol{x}}_m^{\prime}$], *m* is the number of state vectors and ${\boldsymbol{x^{\prime}}}$ is the ensemble anomaly.

Optimal array is determined using the following steps. First, the background error covariance is constructed by using Equation (5). After assimilating *k* observations from a certain location, the corresponding reduction in the background error variance can be calculated using Equation (4). Therefore, we can choose the first optimal observational location that can lead to the maximum error variance reduction of the Niño 3.4 SSTA. Subsequently, the ensemble is updated through the ensemble transform Kalman filter [8] by assimilating the optimal observations:


(6) \begin{eqnarray*}{{{\bf A}}}_{{\bf a}} = {{{\bf A}}}_{{\bf b}}{\left[ {{{\bf I}} \!+\! \frac{1}{{m \!-\! 1}}{{\left( {{{{\bf H}}}^{{\mathrm{opt}}}{{{\bf A}}}_{{\bf b}}} \right)}}^{\mathrm{T}}{{{\bf R}}}^{ - 1}\left( {{{{\bf H}}}^{{\mathrm{opt}}}{{{\bf A}}}_{{\bf b}}} \right)} \!\right]}^{ - 1/2},\end{eqnarray*}

where ${{{\bf A}}}_{{\bf a}}$ is the analysis ensemble. Then we can replace ${{{\bf A}}}_{{\bf b}}$ with ${{{\bf A}}}_{{\bf a}}$, and hence update ${{{\bf P}}}^{{\bf b}}$ to identify the next optimal site. The procedure was repeated until all optimal observational locations were found.

## DATA

The background error covariance was estimated using a time series of monthly ocean temperature anomalies from the EN4 data set [43], which can be downloaded at https://www.metoffice.gov.uk/hadobs/en4/. The data from 1980 to 2020 was used. Although the mooring array could provide high-temporal-resolution sampling, we utilized monthly data to detect optimal sites. This aligns with conventional practices for various ENSO-targeted observation studies, wherein monthly data are typically used [10,41,42,44,45]. The area of the selected temperature data covered the tropical Pacific region from 120°E to 70°W and 20°S to 20°N. We selected 21 levels from 5 to 310 m to show the vertical structure of the temperature properly containing the thermocline. The ocean temperature anomalies were computed by removing their monthly climatological mean.

## Supplementary Material

nwad231_Supplemental_File

## Data Availability

The data underlying this article were accessed from EN4 data set (https://www.metoffice.gov.uk/hadobs/en4/). The derived data generated in this research will be shared on request to the corresponding authors.
