# Supplementary material for: A new ensemble-based targeted observational method and its application in the TPOS 2020
Source: Natl Sci Rev. 2023 Sep 2;10(11):nwad231. doi: 10.1093/nsr/nwad231 (PMC10583287; doi:10.1093/nsr/nwad231)
Supplement: nwad231_Supplemental_File [file nwad231_supplemental_file.pdf]

Supplementary Figure 1.

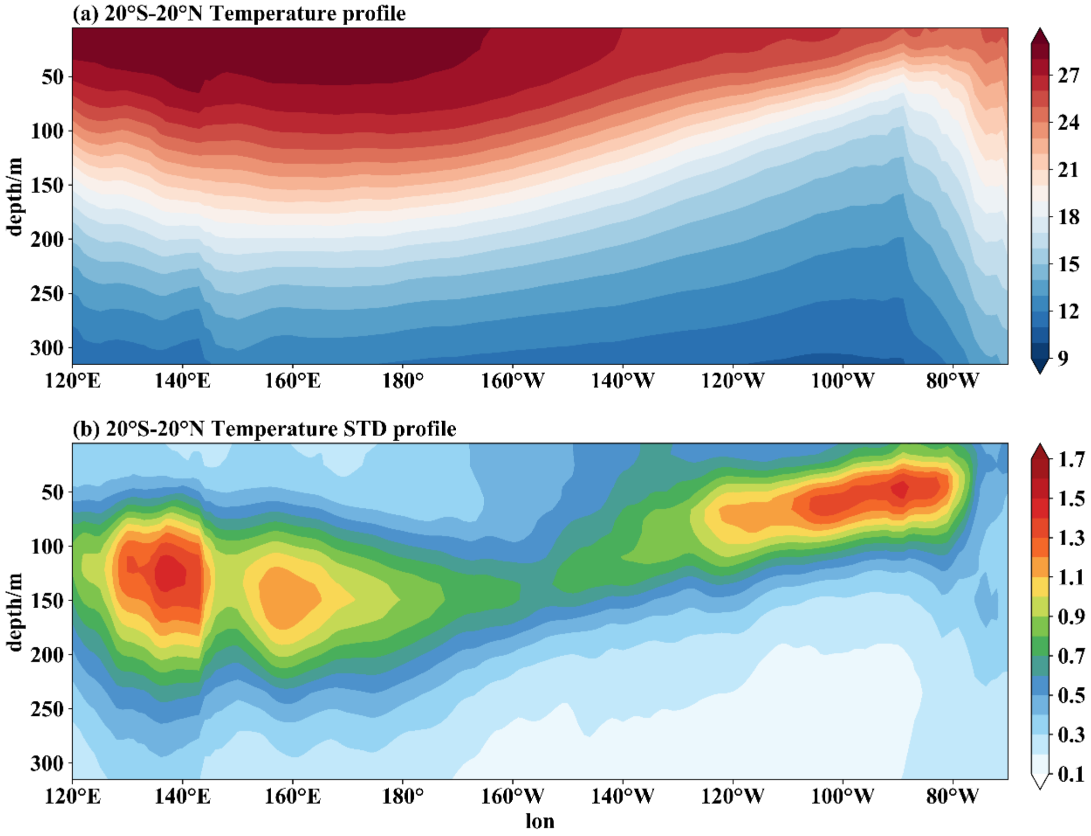

(a) Climatological annual mean and (b) the standard deviation of ocean temperature averaged in 20S-20N from 1980 to 2020.

Supplementary Figure 2.

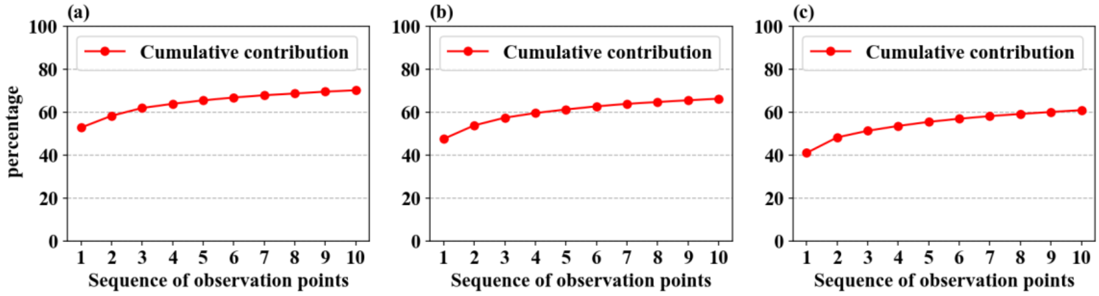

(a-c) The cumulative reduction of the initial error variance of Niño3.4 SSTA using the first n optimal observation locations. This is same as Figure 3b in the manuscript, but with additional white noise to the initial prediction ensemble. The variance of the white noise is 10%, 20% and 30% of the monthly ocean temperature variance in a), b) and c), respectively.
